# First Report of *fusF* Gene in *Staphylococcus kloosii* from Virgin Tropical Soil: Expanding the Ecological Reservoirs of Fusidic Acid Resistance

**DOI:** 10.3390/microorganisms14010197

**Published:** 2026-01-15

**Authors:** Muhammad Haziq Ruzaini Abdullah, Mohd Huzairi Mohd Zainudin, Musheer A. Aljaberi, Noor Azira Binti Abdul Mutalib, Hui-min Neoh, Rukman Awang Hamat

**Affiliations:** 1 Department of Medical Microbiology, Faculty of Medicine and Health Sciences, Universiti Putra Malaysia, Serdang 43400, Malaysia; haziqruzaini98@gmail.com; 2 Institute of Tropical Agriculture and Food Security, Universiti Putra Malaysia, Serdang 43400, Malaysia; mohdhuzairi@upm.edu.my; 3 Department of Science, Franciscus Gasthuis & Vlietland, 3045 PM Rotterdam, The Netherlands; 4 Research Centre Innovations in Care, Rotterdam University of Applied Sciences, 3015 EK Rotterdam, The Netherlands; 5 Department of Internal Medicine, Section Nursing Science, Erasmus University Medical Center (Erasmus MC), 3015 GD Rotterdam, The Netherlands; 6 Department of Food Service and Management, Faculty of Food Science and Technology, Universiti Putra Malaysia, Serdang 43400, Malaysia; n_azira@upm.edu.my; 7 UKM Medical Molecular Biology Institute, Universiti Kebangsaan Malaysia, Kuala Lumpur 56000, Malaysia; hui-min@hctm.ukm.edu.my

**Keywords:** antimicrobial resistance, environmental reservoir, coagulase-negative staphylococci, soil microbiome, *fusF*, One Health

## Abstract

Fusidic acid resistance in *Staphylococcus* spp. has historically been confined to *Staphylococcus ureilyticus*, with limited data on its environmental distribution. This study presents the first detection of the fusidic acid resistance gene *fusF* in *Staphylococcus kloosii* recovered from virgin soil at Kampung Batu 16, Dusun Tua, Hulu Langat, Malaysia. A total of ten *Staphylococcus* isolates were identified using the VITEK^®^2 system with high confidence (97–99%), comprising seven *S. kloosii* and three *S. ureilyticus.* Sequencing of representative isolates further corroborated the species identification. All isolates displayed phenotypic resistance to fusidic acid, while all *S. ureilyticus* (3/3) exhibited multi-drug resistant (MDR) traits and *S. kloosii* (7/7) exhibited non-MDR traits. PCR and sequencing confirmed the presence of *fusF* gene in *S. ureilyticus* (3/3) and *S. kloosii* (3/7). In addition, *fusB* and *fusC* genes were not detected in both species. The phylogenetic analysis (Maximum Likelihood, Tamura–Nei model) revealed high sequence conservation and clustering between *fusF*-positive *S. kloosii* and *S. ureilyticus* soil isolates, suggesting recent horizontal gene transfer between these two related species. The first detection of *fusF* gene in *S. kloosii* from virgin soil signifies the expansion of the ecological and host range beyond *S. ureilyticus*, establishes virgin soil as a potential antimicrobial resistance (AMR) reservoir, and underscores the One Health risks of resistance dissemination from environmental staphylococci. This baseline study highlights the importance of early AMR surveillance in tropical environments prior to agricultural development.

## 1. Introduction

Soil ecosystems comprise complex biological matrices that host various microbial communities vital for nutrient cycling, decomposition of organic matter, and overall soil health. Within soil communities, bacteria play a crucial role in facilitating biochemical processes and maintaining ecological balance [1,2,3]. *Staphylococcus* spp. form part of the soil microbiome, particularly in environments affected by human or animal activity [4,5]. These bacteria are not only widespread in soils but also possess One Health significance, as their various species are implicated in the transmission of antimicrobial resistance genes (ARGs) among humans, animals, and the environment, orchestrated by mobile genetic elements (MGEs) [6,7,8,9,10,11]. The discovery of methicillin-resistant *Staphylococcus aureus* (MRSA) in tropical soils of Hilo Bay, Hawaii, by Gerken, Wiegner, and Economy [4] has sparked interest among scientists in the concerning possibility that soils may act as unrecognised reservoirs for clinically significant *Staphylococcus* species and their associated ARGs.

Meanwhile, fusidic acid, which has been commonly used as a single topical agent for treating staphylococcal infections in humans and animals for many decades, may also exert continuous selective pressure for the possibility of spillover of fusidic acid residues into the soil environment [12,13]. Fusidic acid inhibits bacterial protein synthesis by binding to the elongation factor G (EF-G) [14]. Resistance to this antibiotic has been recognized among clinical staphylococci, where factors for the selective pressure are mainly restricted to clinical settings such as heavy topical use of fusidic acid [15,16], prolonged use of fusidic acid in chronic dermatoses [17], and inappropriate use of fusidic acid as a mono-therapeutic agent for skin conditions [18]. In addition, mechanisms of resistance of fusidic acid in staphylococci have been identified due to a single point mutation of *fusA* gene, which encodes for EF-G and multiple mutations of *fusB*, *fusC*, and *fusD* genes encoding for FusB-family proteins [19,20]. The *fusF* gene, which also belongs to the FusB family, was first identified in *S. cohnii*, which exhibited low fusidic acid resistance. Further investigation revealed that expression of the *fusF* gene in *S. aureus* demonstrated high minimum inhibitory concentrations (MICs) of fusidic acid (16 mg/L) among these isolates, confirming that the *fusF* gene contributes to fusidic acid resistance [21]. Surprisingly, coagulase-negative staphylococci (CoNS) often acquire a wider carriage of fusidic-resistance determinants than *S. aureus* [6,22]. For instance, *fusB*-mediated resistance is frequently reported in species such as *S. epidermidis* and *S. haemolyticus*, positioning CoNS as important reservoirs for fusidic-acid-resistance genes [18,19]. In addition, CoNS detected on skin from healthy volunteers have been shown to exhibit fusidic resistance, establishing their occurrence outside the clinical settings [23,24].

However, studies on the presence and type of genetic determinants of fusidic acid (FA)-resistant CoNS in undisturbed (virgin) soils remain unexplored. Recent findings suggest that the *fusF* gene is intrinsically found in all *S. cohnii* and *S. urealyticus* species isolated from German dairy farms [25]. It is plausible to explore whether soil staphylococci have fusidic-acid-resistance determinants, which may be potentially driven by clinical or agricultural exposures. Their presence in virgin or undisturbed soils that are later converted to farmland raises concerns about contamination of vegetables, particularly in organic or low-input farming systems that lack chemical sanitization. Moreover, the genus is well known for its biofilm-forming ability, environmental resilience, and capacity to transmit resistance genes to other soil- or plant-associated microbes via horizontal gene transfer, potentially amplifying public health risks when such produce enters the food supply chain [26,27].

Taken together, these considerations highlight the need for early and prompt surveillance of environmental staphylococci, especially in minimally disturbed virgin soil, to anticipate potential pathways for antimicrobial resistance (AMR) dissemination prior to agricultural or residential development. Such surveillance aligns with the One Health approach by bridging environmental, animal, and human health perspectives to mitigate AMR risk in agricultural practices. Hence, the study aimed to investigate the presence of fusidic acid resistance genes among environmental staphylococci in a Malaysian virgin soil. We hypothesize that environmental staphylococci isolated from Malaysian virgin soil carry fusidic acid resistance determinants and demonstrate reduced susceptibility to fusidic acid, despite minimal anthropogenic influence.

## 2. Materials and Methods

### 2.1. Study Site

**Figure 1:**
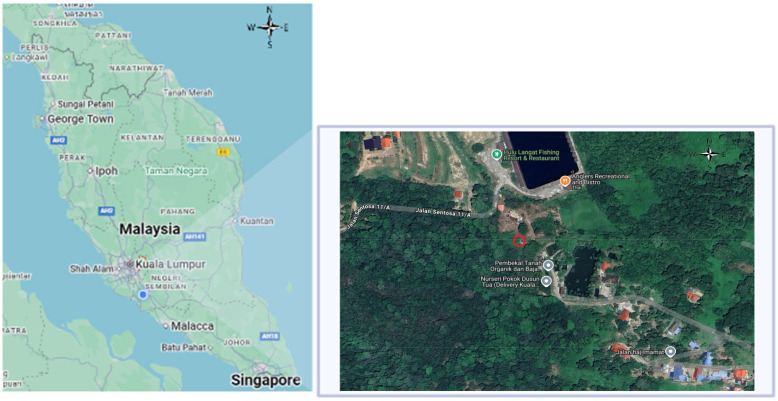
Sampling site of the study area in Kampung Batu 16, Dusun Tua, Hulu Langat, Selangor (see red circle). The coordinate of the sampling site is 3°8.6566′ N, 101°50.0792′ E.

The study site was located at an undisturbed, virgin land in Dusun Tua, Hulu Langat, Selangor (3°8.6566′ N, 101°50.0792′ E). The location of the land is illustrated in Figure 1. It has a tropical rainforest climate, with a mean annual temperature of 28.4 °C and an average annual precipitation of 2455 mm. To confirm the sampling site’s classification as virgin soil, an on-site visual assessment was conducted prior to sampling. The site showed no signs of anthropogenic disturbance and activity, such as agricultural cultivation, livestock farming within a 10 km radius, built infrastructure, or chemical inputs. This visual assessment included evaluating the absence of tilled rows, crop residues, manure application, irrigation systems, or synthetic materials. Additionally, informal interviews with the landowner indicated that the area had remained uncultivated and unmanaged for at least the past 10 years.

### 2.2. Soil Sampling

In July 2019, soil samples were collected from a virgin land dominated by annual grasses at Kampung Batu 16, Dusun Tua. Sampling followed a grid-based approach adapted from previous publications, with slight modifications to suit the relatively small study site area (16.5 m × 5 m) [28,29]. Specifically, the site was subdivided into eight equal-sized plots (3.0 m × 1.5 m each) to enable systematic spatial coverage while maintaining adequate resolution for detecting local heterogeneity in the virgin soil. Next, five sampling points were evenly distributed across the plot prior to fertilization to establish baseline homogeneity. At each point, five soil subsamples were collected aseptically using a sterile portable handheld t-sampler, each taken from 0–20 cm, capturing both the surface and subsurface soil layers relevant to microbial activity. This depth was selected to encompass the active soil zone and to include microbial communities residing beneath the topsoil. Grass and weeds were removed prior to soil sampling.

The subsamples from each sampling point were pooled and homogenized in a sterile stomacher bag (Gosselin, Saint-Germain-en-Laye, France) to form one composite sample per plot, resulting in a total of eight composite soil samples. This design enabled representative sampling across the entire site while minimizing local heterogeneity. Sampling was conducted in the early morning (between 7:30 and 9:00 AM) to minimize diurnal variation in soil temperature and microbial activity, which can influence both community composition and metabolic states [30]. The bags were sealed immediately, labelled, and kept on ice until arrival at the laboratory for processing. All samples were stored at 4 °C and processed within 24 h to preserve microbial viability.

### 2.3. Isolation of Pure Bacterial Culture

Serial dilutions (10^−1^ to 10^−5^) of bacteria were prepared in Luria–Bertani (LB) broth following homogenization of 50 g of soil in sterile LB broth to obtain the primary suspension. From each tube, 100 μL of diluted samples were spread onto mannitol salt agar (MSA) (Isolab, Shah Alam, Malaysia) and incubated at 37 °C for 24 h. Presumptive staphylococcal colonies were identified based on their ability to grow on MSA, in which yellow discolouration will be observed around the mannitol-fermenting colonies on the medium, while non-fermenters will remain pink in colour [31]. Positive (*S. aureus* ATCC 25923) and negative (*S. epidermidis* ATCC 12228) controls were used for comparison. To ensure accuracy and reproducibility, only plates yielding 30–300 colony-forming units (CFU) were considered for further examination, consistent with standard microbiological enumeration guidelines [32]. From these plates, three predominant, well-separated single colonies were selected, as such colonies are more likely to represent the ecologically dominant and clinically relevant members of the microbial community while ensuring culture purity. Selected colonies were streaked onto Columbia agar with 5% sheep blood (Isolab, Malaysia) to obtain pure isolates for downstream identification. Each isolate was labelled with an alphanumeric identifier in the format of P#-D#-C#, denoting the plot number (P), dilution level (D), and colony number picked from the respective plate (C).

### 2.4. Bacterial Identification and Antimicrobial Susceptibility Testing via VITEK^®^2 System

Pure cultures were screened using morphological and biochemical tests, including Gram staining and the catalase test, prior to subsequent identification. This preliminary screening confirmed that the isolates were pure, correctly characterized, and free from contamination, thereby enhancing the reliability of subsequent identification steps.

Pure bacterial colonies were then suspended in sterile saline (0.45% NaCl) and adjusted to a turbidity equivalent to the McFarland standard recommended for the VITEK^®^2 system (v9.11.0), specifically 0.5 McFarland (approximately 1 × 10^8^ CFU/mL) (bioMérieux, Marcy-l’Étoile, France). For species identification, Gram-positive (GP) identification cards were inoculated according to the manufacturer’s instructions and incubated at 35.5 ± 1.0 °C. To further establish the species identification, DNA extracts from a few representative isolates were prepared (see Section 2.6) and sent to Apical Scientific (Apical Scientific, Seri Kembangan, Selangor, Malaysia) for sequencing. Antimicrobial susceptibility testing (AST) was performed using the VITEK^®^2 Gram-positive Susceptibility Card. Minimum inhibitory concentrations (MICs) were automatically determined and interpreted according to established international guidelines, including those of the Clinical and Laboratory Standards Institute [33].

Antibiotic susceptibility testing was performed automatically by the VITEK^®^2 system using the microbroth dilution method. The antibiotic panels involved several antimicrobial classes such as penicillins (benzylpenicillin, cloxacillin, oxacillin); β-lactam/β-lactamase inhibitor combinations (amoxicillin/clavulanic acid, ampicillin/sulbactam, piperacillin/tazobactam); cephalosporins (cefuroxime, cefoperazone, cefotaxime, ceftriaxone, cefepime, ceftaroline); carbapenems (ertapenem, imipenem, meropenem); aminoglycosides (gentamicin); fluoroquinolones (ciprofloxacin, levofloxacin, moxifloxacin); macrolides (azithromycin, erythromycin); lincosamides (clindamycin, including inducible clindamycin resistance assay); glycopeptides (vancomycin, teicoplanin); tetracyclines (tetracycline, tigecycline); and other agents including fosfomycin, fusidic acid, rifampicin, trimethoprim–sulfamethoxazole, and the cefoxitin disc. However, a few antibiotics could not be interpreted by the CLSI guidelines for *Staphylococcus* spp. (e.g., carbapenems, fosfomycin, and high-level aminoglycosides) were excluded from the final susceptibility table to avoid misinterpretation [33]. Only antimicrobial agents with valid sensitive (S)/resistant (R) breakpoints were reported. Multidrug-resistant (MDR) bacteria are defined as organisms resistant to at least one agent in at least three antimicrobial classes [34].

### 2.5. Detection of Antibiotic Resistance Genes

PCR amplification was performed using a PCR thermocycler (Bio-Rad, Hercules, CA, USA). In the present study, *fusB*, *fusC*, and *fusF* genes were targeted for amplification. The sequences of the primers used were sourced from previous studies [21,35]. All primers were synthesized by Apical Scientific (Apical Scientific, Selangor, Malaysia), and their information is available in the Supplementary material (Table S1).

The mix reaction was performed with 1× of MyTaq™ Red Mix (Bioline, London, UK), 0.4 μM for both forward and reverse primers, 10–200 ng of DNA template, and deionized water was used to reach a final volume of 25 μL. Both positive (previously confirmed *fusB-* and *fusC*-positive *S. epidermidis*) and negative controls (without template DNA) were run in triplicate. Each reaction was run in triplicate with the following cycling parameters: 1 cycle at 95 °C for 1 min (initial denaturation), followed by 30 cycles of 95 °C for 15 s (denaturation), 50 °C for 15 s (annealing), and 72 °C for 10 s (extension). The PCR products were verified by Sanger sequencing to confirm their identities (see Section 2.6). Since we could not obtain a positive control strain of the *fusF* gene, the established forward and reverse primers of the *fusF* gene were used in the protocol according to Chen, Hung, Lin, Tsai, Chiu, Hsueh, and Teng [21], and the PCR product was sequenced to observe the similarity index.

### 2.6. DNA Sequencing and Sequence Analysis

PCR amplicons obtained from the amplification of 16S rRNA and *fusF* genes were verified through bidirectional Sanger sequencing by a commercial service provider (Apical Scientific, Selangor, Malaysia). Raw sequence chromatograms were quality checked, assembled into consensus sequences, and compared against reference sequences available in the NCBI GenBank database using the BLASTn algorithm to confirm species identity and *fusF* gene assignment (National Library of Medicine; accessed at https://www.ncbi.nlm.nih.gov/nucleotide/) (accessed on 15 October 2025).

### 2.7. Phylogenetic Analysis

To further elucidate the evolutionary relationship of the detected *fusF* sequences, molecular phylogenetic analysis was performed. The National Center for Biotechnology Information (NCBI) Nucleotide database (National Library of Medicine; accessed at https://www.ncbi.nlm.nih.gov/nucleotide/) (accessed on 30 October 2025) was used to align representative *fusF* gene sequences from our study with two *S. ureilyticus fusF* reference sequences retrieved from GenBank (designated as Control 1 (NG_047903.1) and Control 2 (NG_047904.1)) and with one *S. aureus fusC* gene sequence included as an outgroup. Details of the published sequences retrieved from GenBank and used for phylogenetic analysis are provided in the Supplementary Material (Table S2). Multiple sequence alignment was performed using ClustalW v2.1 (https://www.clustal.org/clustal2/) (accessed on 12 November 2025) with default parameters.

Phylogenetic trees were constructed using the Maximum Likelihood (ML) method based on the Tamura & Nei (1993) model as implemented in MEGA11 (v11.0.13; https://www.megasoftware.net/) (accessed on 12 November 2025) [36,37]. The Tamura–Nei model was selected as it accounts for unequal nucleotide frequencies and transition/transversion rate bias, making it suitable for analyzing gene-level bacterial sequences. Initial tree(s) for the heuristic search were obtained automatically by applying Neighbour-Join and BioNJ algorithms to a matrix of pairwise distances estimated using the Maximum Composite Likelihood (MCL) approach. The reliability of the resulting tree topology was assessed using bootstrap analysis with 1000 replicates. Only bootstrap values above 70% were considered to provide moderate to strong support for branching patterns. The final tree was visualized and annotated with species names and isolate codes for comparison with environmental and reference strains.

The ML method was selected for its statistical robustness, particularly for modeling site-specific nucleotide substitution patterns and detecting subtle evolutionary divergence among closely related sequences. This method is especially appropriate for assessing potential horizontal gene transfer (HGT) of resistance genes, such as *fusF*, between environmental and reference isolates.

## 3. Results

### 3.1. Phenotypic Findings of the Isolates

A total of 10 *Staphylococcus* isolates were identified by the VITEK®2 system with high confidence levels (97–99%), comprising predominantly *S. kloosii* (7/10) and *S. ureilyticus* (3/10) (Table 1). All *S. kloosii* isolates (100%) exhibited dual resistance to fusidic acid and tetracycline. Broader resistance profiles were observed in three *S. ureilyticus* isolates (P3-D3-C1, P1-D3-C1, and P7-D4-C2), which were resistant to azithromycin, erythromycin, clindamycin, and fusidic acid. All these isolates were classified as multidrug-resistant (MDR) strains, whereas the remaining isolates were categorized as non-MDR strains. Notably, universal resistance to fusidic acid was observed across all isolates. The antibiotic susceptibility patterns are provided in the Supplementary File, Table S4.

**Figure 2:**
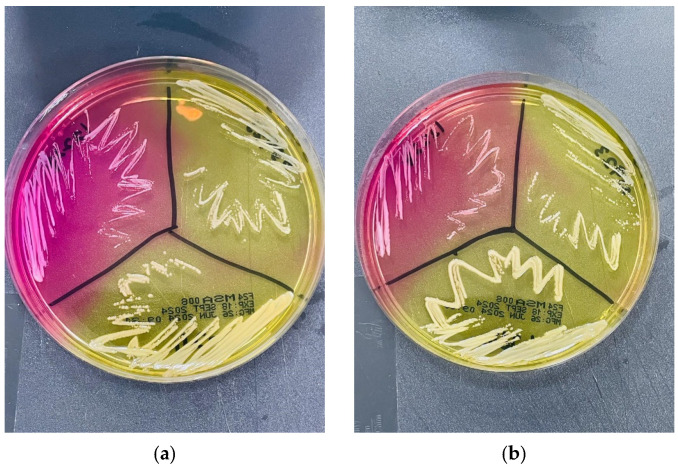
Growth of reference strains and environmental staphylococci on mannitol salt agar (MSA). On both MSA plates (**a**,**b**), the left quadrant shows *Staphylococcus epidermidis* ATCC 12228, the right quadrant shows *Staphylococcus aureus* ATCC 25923, and the middle quadrant shows *Staphylococcus kloosii* (P3-D4-C2) and *Staphylococcus ureilyticus* (P1-D3-C1), respectively. Both *S. kloosii* (**a**) and *S. ureilyticus* (**b**) produced yellow discoloration around their colonies resembling *S. aureus*, underscoring the limitation of morphological-based identification for these environmental staphylococci.

Yellow discolouration was observed on mannitol salt agar (MSA) around *S. kloosii, S. ureilyticus*, and *S. aureus* ATCC 25923 (positive control) colonies, while the medium retained its pink colour around *S. epidermidis* ATCC 12228 (negative control) colonies (Figure 2).

### 3.2. Genotypic Findings of the Isolates

Among the 10 fusidic-resistant environmental staphylococci, six isolates (*S. ureilyticus* C1, C2, and C3 and *S. kloosii* K1, K2, and K4) tested positive for the *fusF* gene by PCR. The corresponding *fusF* amplicons shared 100% sequence identity with reference *fusF* sequences (Table 2). Agarose gel electrophoresis (1.5%) results and BLAST-based sequence identity (https://www.ncbi.nlm.nih.gov/nucleotide/) (accessed on 12 November 2025) for the *fusF* amplicons are provided in Supplementary Materials Figure S1 and Table S1. The *fusF* gene was not detected in the remaining four isolates (*S. kloosii* K3, K5, K6, and K7). No *fusB-* and *fusC*-positive strains were detected among all isolates. To further validate species identification, partial 16S rRNA gene sequencing was performed for representative isolates. BLASTn (https://www.ncbi.nlm.nih.gov/nucleotide/) (accessed on 8 December 2025) analysis against the NCBI GenBank database showed high sequence similarity (≥99.9% identity, 100% query coverage, E-value = 0.0) to reference sequences of *S. ureilyticus* and *S. kloosii*. These results further supported species identification of *S. ureilyticus* and *S. kloosii* as identified by the VITEK®2 system. The nucleotide sequences generated in the present study have been deposited in GenBank. The 16S rRNA sequences are available under accession numbers PX765221, PX765222, and PX765223. As for the *fusF* gene sequences, the accession numbers PX828997, PX828998, PX828999, PX829000, PX829001, and PX829002 are provided (Supplementary Materials, Tables S2 and S3).

**Figure 3:**
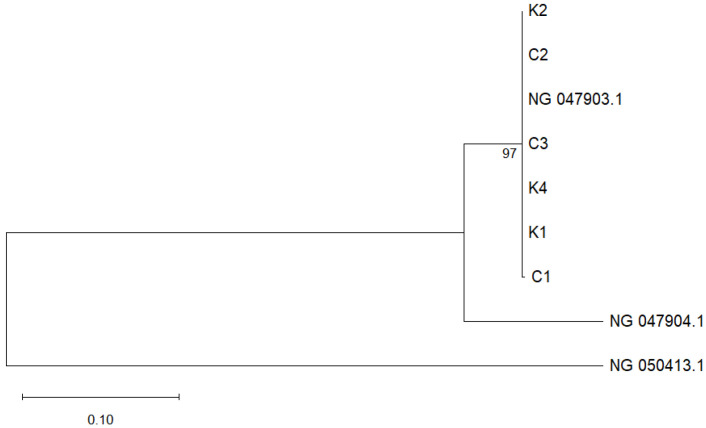
Phylogenetic tree based on *fusF* gene sequences constructed using the maximum-likelihood method in MEGA 12. Bootstrap values (≥50%) from 1000 replications are indicated at branch nodes. The *S. kloosii* and *S. ureilyticus* isolates obtained in this study are labeled K1, K2, K4, C1, C2 and C3, respectively, while *fusF* reference sequence genes of *S. ureilyticus* NG_047903.1 and NG_047904.1 are retrieved from the GenBank. A reference sequence of *S. aureus fusC* gene (NG_050413.1) is used as an outgroup. The scale bar represents the number of nucleotide substitutions per site.

Given the absence of *fusB* and *fusC* among the isolates, subsequent phylogenetic analysis focused on the *fusF* gene sequences was performed. Maximum likelihood phylogenetic analysis revealed a clear and well-supported clustering pattern among the environmental isolates and reference strains (Figure 3). All environmental isolates from this study (K1, K2, K4, C1, C2, and C3) grouped with NG_047903.1, with strong bootstrap support of 97%. The reference sequence NG_047904.1 branched as a sister lineage outside the main cluster. Notably, NG_050413.1, representing the *fusC* sequence from *S. aureus*, branched distantly from the *fusF* cluster, thereby confirming its role as an outgroup.

## 4. Discussion

Increasing reports have been observed in studies involving staphylococci that harbor AMR-genes via MGEs on hospital surfaces, in the community [23], in residential environments [38], and in animals [39]. In our study, a universal phenotypic resistance to fusidic acid (FA) across all 10 environmental staphylococci was observed, despite the absence of detectable FA resistance genes in four *S. kloosii* isolates (see Table 2). This observation is striking given that FA resistance has historically been considered rare, with reports largely confined to *S. aureus* and a limited number of CoNS in clinical contexts [35,40]. Interestingly, *fusF* gene was consistently detected in all three isolates of *S. ureilyticus* (formerly known as *S*. *cohnii* subsp. *urealyticus*) in our study. However, we could not determine whether the *fusF* gene in our isolates is chromosomally encoded (intrinsic resistance) or plasmid-mediated (acquired resistance). Nonetheless, *fusF* gene was also discovered in all *S. ureilyticus* and *S. cohnii* isolates recovered from the farm and clinical settings [21,25]. *FusF* is a novel FusB-family gene first detected in *S. ureilyticus* clinical isolates and has since spread widely among CoNS [21].

Although the VITEK^®^2 system is widely used to identify clinically relevant staphylococci, its reference database is largely based on clinical and host-associated isolates. For this reason, partial 16S rRNA gene PCR and sequencing were performed to confirm the species assignments of representative environmental isolates recovered from virgin soil in the present study. Environmental staphylococci from non-clinical niches, including undisturbed soil, may also display phenotypic variation that can reduce the reliability of biochemical-based identification [41]. In terms of performance, the VITEK®2 system correctly identifies 54.3% of isolates at the species level and 77.1% at the genus level, whereas 16S rRNA gene sequencing achieves 94.3% and 100% accuracy for species-level identification, respectively. This difference highlights the impact of database coverage on identification performance [42]. Consistent with large-scale evaluations, automated systems tend to perform well on common clinical taxa but may yield discordant or unresolved results for rare or atypical organisms, necessitating molecular confirmation [43]. The 16S rRNA gene sequences of the representative isolates showed very high similarity to reference sequences (99.93–100%). It can be concluded that 16S rRNA gene sequencing provided a robust taxonomic basis for interpreting the distribution of the *fusF* gene among soil-associated isolates in the present study. These findings support that *fusF* is genuinely present in *S. kloosii* and *S. ureilyticus* from a non-clinical environment, rather than reflecting database-driven misidentification.

To the best of our knowledge, this is the first detection of *fusF* gene in *S. kloosii* isolates (three of six) recovered from undisturbed soil. The inconsistency in *fusF* gene detection among *S. kloosii* isolates in our study suggests that *fusF* may be sporadically acquired from other *Staphylococcus* spp. via mobile genetic elements (MGEs) such as plasmids and transposons. It has been demonstrated that transferable plasmids were responsible for the dissemination of *fusB* and *fusC* genes in *S. aureus* and CoNS [21,44]. Moreover, *fusF* gene was detected in both *S. cohnii* and *S. ureilyticus* isolates from the same farm environment in Germany, and it was suggested that the spread of *fusF* gene may occur via horizontal gene transfer between these two species [25]. A similar explanation can be deduced for some of *S. kloosii* isolates in our study, as *S. ureilyticus* can transmit *fusF* gene to its closely related species when suitable environmental conditions are able to facilitate the conjugative transfer.

This is further supported by the maximum likelihood phylogenetic analysis of *fusF* gene sequences in which three *S. kloosii* (K1, K2, K4) and three *S. ureilyticus* (C1, C2, C3) formed a single, well-supported monophyletic cluster (Figure 3). The tight cluster (bootstrap 97%) and short branches may indicate a recent *fusF* gene sharing event among these environmental staphylococci. The high sequence similarity of *fusF* sequences from *S. kloosii*-a species that is not previously known to harbour *fusF* gene, could possibly suggest a recent horizontal gene transfer (HGT) event by *S. ureilyticus* (a natural *fusF* gene donor/reservoir species) in the same virgin soil. Gene flow via HGT is extensive among CoNS, including *S. ureilyticus*, and resistance genes can be shared among inter- or intra-species [45]. This is also in line with a study proposing that recent HGT or divergence of *fusF* variants is occurring among *S. cohnii* and *S. urealyticus* on the same farm [25]. Meanwhile, the placement of NG_047904.1 as a sister lineage outside the main cluster further suggests ongoing diversification within the *fusF* lineage. The distant positioning of the *fusC* reference sequence (NG_047903.1) further supports the phylogenetic distinction between *fusC* and *fusF* lineages.

It is well known that following anthropogenic activities, FA residues can exert selective pressure, promoting the emergence and persistence of FA resistance in *Staphylococcus* spp. [22]. However, it is plausible to highlight that the detection of *fusF* gene in a subset of *S. kloosii* isolates from undisturbed soil in our study may suggest that non-anthropogenic selection mechanisms should also be considered. This observation could be explained by the natural presence of AMR genes (resistomes) in soil or by fusidic-acid-producing soil microbiomes, such as *Fusidium coccineum* (formerly known as *Acremonium fusidioides*), even in the absence of prior human or veterinary antibiotic exposure. For instance, fungal secondary metabolites such as fusidic acid, produced by *F. coccineum* may create localized soil micro-niches that exert intermittent selective exposures on surrounding soil microbiomes. Over time, this microenvironment could promote the persistence of *fusF*-carrying bacteria and accelerate the spread of *fusF* gene [46,47,48,49]. It may also reflect the persistence and dynamic exchange of AMR genes, including *fusF* within soil microbiota via HGT [50]. Moreover, this broad diversity of AMR genes existed before antibiotics were used for humans on animals [51]. Nonetheless, the discovery of *fusF* gene in both *S. ureilyticus* and *S. kloosii* recovered from pristine soil suggests that FA resistance may be more environmentally widespread than previously recognized.

In our study, FusB-family genes (*fusF*, *fusB,* and *fusC*) were not detected in the remaining four of eight *S. kloosii* isolates, and *fusB* and *fusC* genes were also not detected in all *S. ureilyticus* isolates. Although a target protection mechanism conferred by FusB-family genes is recognized as the most predominant FA resistance [22,52,53], it is plausible that other resistance mechanisms may be involved and yet to be fully discovered among environmental staphylococci [54,55]. Interestingly, no FusB-family genes (including *fusF*) were detected in *S. kloosii* isolates recovered from ready-to-eat foods, even though these strains exhibited low-level FA resistance [56]. This finding further emphasizes the likelihood that *S. kloosii* isolates may potentially confer alternative resistance determinants beyond the FusB family. Thus, robust genomic and functional studies are warranted to elucidate the underlying mechanisms of resistance in the future.

Despite the small sample size in our study, all *S. ureilyticus* isolates exhibited MDR traits, which is consistent with other studies. For example, animal-derived *S. ureilyticus* and *S. cohnii* isolates have been reported to harbor various AMR genes and exhibit resistance to multiple antibiotic classes [57,58,59]. In clinical settings, *S. ureilyticus* is associated with infections in immunocompromised patients [60]. Interestingly, a recent genomic study of *S. ureilyticus* (MUWRP0921 strain) isolated from the urine sample of an adult female Ugandan revealed its MDR traits to various classes of antibiotics [61]. MDR traits of *S. ureilyticus* obtained from virgin soil in our study may underscore the importance of surveillance of this species via the One Health approach. In our study, all *S. kloosii* isolates exhibited non-MDR traits. Previous findings on the MDR traits of *S. kloosii* have yielded conflicting results: two studies reported isolates with non-MDR profiles [62,63], whereas only one study reported strains with MDR profiles [64].

Despite the novel insights, the study has several limitations. Firstly, the soil sampling was restricted to a relatively small site and a single time point, which may not capture the broader spatiotemporal variability of staphylococcal populations in tropical soils. Secondly, resistance screening was limited to *fusB*, *fusC,* and *fusF*, leaving other *fus* gene variants unexamined, limiting the full characterization of fusidic acid resistance mechanisms. In addition, the genetic basis of other phenotypic resistance observed (e.g., tetracycline, macrolides, lincosamides) was not explored, which may have provided deeper insights into the resistome of soil staphylococci. Future work should expand sampling across sites and seasons and apply whole genomes to clarify the diversity and mobility of resistance determinants in virgin soil. Finally, rapid detection methods such as LAMP can be utilized to monitor resistance genes in the environment; hence, the One Health approach can be implemented across different ecosystems [9].

## 5. Conclusions

This study provides the first evidence of the *fusF* gene in *Staphylococcus kloosii* isolated from virgin soil in Hulu Langat, Malaysia. However, *fusF* may not be the primary determinant of FA resistance in *S. kloosii,* and other potential FA resistance determinants may be involved. *S. ureilyticus* appears to serve as a potential donor/reservoir of the *fusF* gene for *S. kloosii* based on the phylogenetic analysis. These findings suggest that, despite a lack of exposure to anthropogenic activities in virgin soil, the persistent and dynamic exchange of AMR genes persists within the soil microbiota, underscoring the role of minimally disturbed soils as hidden reservoirs of AMR. Strengthening One Health surveillance frameworks and guiding strategies to mitigate AMR risks at the human–environment interface is urgently warranted.

## Figures and Tables

**Table 1:** Bacteria identification and antimicrobial resistance profiles of isolates identified by the VITEK^®^ system.

Isolate ID	Species	VITEK2^®^ ID Confidence Level	Resistance Patterns ^†^	MDR Status *
P1-D3-C1	*S. ureilyticus*	99%	AZM, ERY, CLI ^δ^, FA	●
P1-D4-C1	*S. kloosii*	97%	FA, TET	○
P2-D3-C1	*S. kloosii*	99%	FA, TET	○
P1-D4-C1	*S. kloosii*	99%	FA, TET	○
P3-D3-C1	*S. ureilyticus*	99%	AZM, ERY, CLI ^δ^, FA	●
P3-D4-C1	*S. kloosii*	97%	FA, TET	○
P3-D4-C2	*S. kloosii*	97%	FA, TET	○
P6-D3-C1	*S. kloosii*	97%	FA, TET	○
P7-D4-C1	*S. kloosii*	99%	FA, TET	○
P7-D4-C2	*S. ureilyticus*	99%	AZM, ERY, CLI ^δ^, FA	●

^†^ resistance by the VITEK^®^2 system (bioMérieux, Marcy-l’Étoile, France) using the MIC method; * MDR status is signified by the symbols (●) and (○) indicating multidrug-resistant and non-multidrug-resistant isolates, respectively; ^δ^ inducible clindamycin resistance; AZM (azithromycin), ERY (erythromycin), CLI (clindamycin), FA (fusidic acid), TET (tetracycline).

**Table 2:** The distribution of *fusB*, *fusC* and *fusF* genes and BLAST similarity results among 10 environmental staphylococci in the present study.

Species ^δ^	PCR ID	Fusidic Acid Resistance ^†^	*fusB*	*fusC*	*fusF*	BLAST Identity of *fusF*
*S. ureilyticus*	C1	R	−	−	+	100%
*S. kloosii*	K1	R	−	−	+	100%
*S. kloosii*	K2	R	−	−	+	100%
*S. kloosii*	K3	R	−	−	−	−
*S. ureilyticus*	C2	R	−	−	+	100%
*S. kloosii*	K4	R	−	−	+	100%
*S. kloosii*	K5	R	−	−	−	−
*S. kloosii*	K6	R	−	−	−	−
*S. kloosii*	K7	R	−	−	−	−
*S. ureilyticus*	C3	R	−	−	+	100%

^†^ resistance by the VITEK^®^2 system (bioMérieux, Marcy-l’Étoile, France) using the MIC method; +/− indicates the presence or absence of PCR products by the 1.5% gel electrophoresis; ^δ^ both species are further confirmed by sequencing; R = Resistant.

## Data Availability

The raw data supporting the conclusions of this article will be made available by the authors on request.

## Supplementary Materials

The following supporting information can be downloaded at https://www.mdpi.com/article/10.3390/microorganisms14010197/s1, Figure S1: Representative gel images of 16S rRNA and *fusF*-positive amplicons obtained in the present study. Table S1: Information about the oligonucleotide primers used in the present study; Table S2: Information on the BLAST identity of the *fusF* sequences obtained in the present study; Table S3: Information about the BLAST identity of the partial 16S rRNA sequences obtained in the present study. Table S4: Information on the nucleotide sequences of the fusidic acid resistant genes retrieved from GenBank database used in the construction of the phylogenetic tree. Table S5: Antimicrobial susceptibility testing (AST) profiles (MIC values and categorical interpretations) of 10 environmental *Staphylococcus* isolates tested using the VITEK^®^2 panel.

## Abbreviations

The following abbreviations are used in this manuscript:

UPM	Universiti Putra Malaysia
ATCC	American Type Culture Collection
et al.	et alia (and others)
°	Degree
°C	Degree Celsius
%	Percentage
m	Metre
mm	Millimetre
ng	Nanogram
μL	Microlitre
h	Hour
s	Second
bp	Base pair
spp.	Several species
subsp.	Subspecies
