# Supplementary material for: First Report of fusF Gene in Staphylococcus kloosii from Virgin Tropical Soil: Expanding the Ecological Reservoirs of Fusidic Acid Resistance
Source: Microorganisms. 2026 Jan 15;14(1):197. doi: 10.3390/microorganisms14010197 (PMC12843955; doi:10.3390/microorganisms14010197)
Supplement: Supplementary file 1 [file microorganisms-14-00197-s001.zip › R4 Revised microorganisms-4005169-supplementary filesEDIT9.1.pdf]

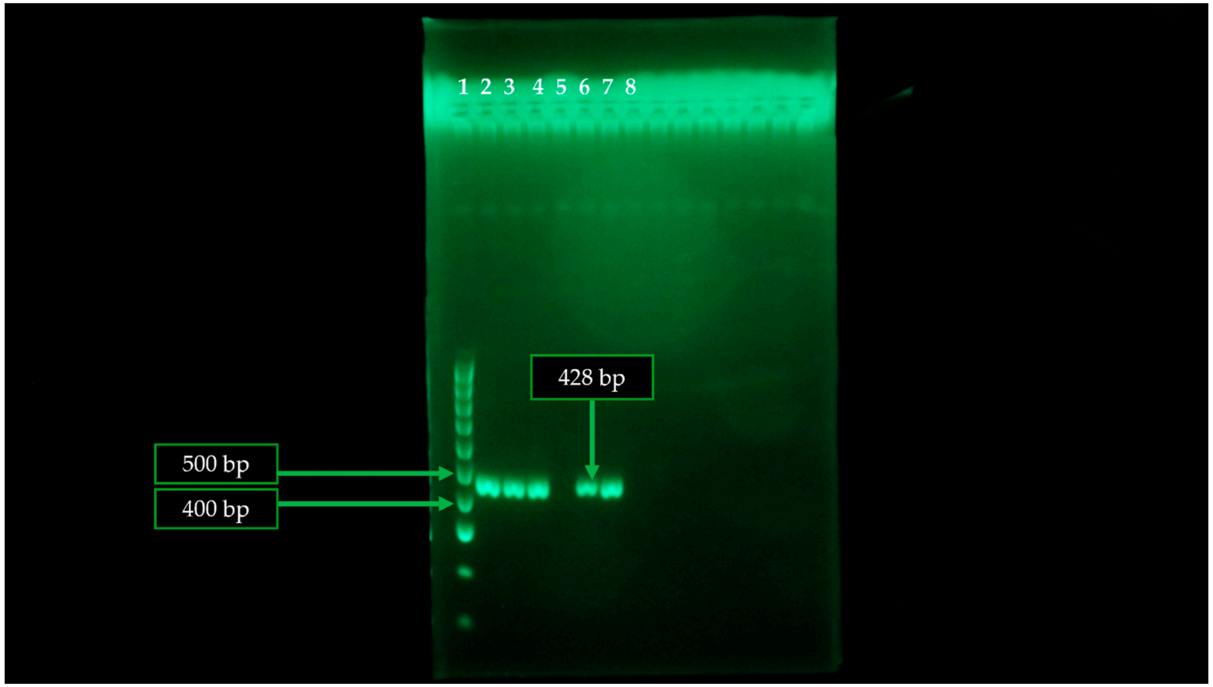

Figure S1. Representative gel images of 16S rRNA and *fusF*-positive amplicons obtained in the present study.

Note: Agarose gel electrophoresis of *fusF* gene PCR amplicons in representative six *Staphylococcus* isolates. Positive amplification at the expected size (~428 bp) was observed in lanes 2–4 and 6–7, corresponding to isolates C1, K1, K2, C2, and K4, respectively. No amplification was detected in lane 8 (K3) or in the no-template control (NTC). Lane 1 contains a 1 kb DNA ladder (Bioline, UK).

Table S1. Information about the oligonucleotide primers used in the present study.

Primer name	Sequence (5' to 3')	Size of PCR product (bp)	Reference
27F	AGAGTTTGATCCTGGCTCAG	1499	

1492R	TACGGYTACCTTGTTACGACTT		Bennasar et al., 1996
fusB-Forward	CCGTCAAAG TTATTCAATCG	491	Chen et al., 2010
fusB-Reverse	ACAATGAAT GCTATCTCGACA		
fusC-Forward	GGACTTTATTACATCGATTGAC	411	
fusC-Reverse	CTGTCATAACAAATGTAATCTCC		
fusF-Forward	CTAAAATAGACATTTATCAGCAG	428	Chen et al., 2015
fusF-Reverse	GGTATATTG TCCATCACCAG		

Table S2. Information about the BLAST identity of the *fusF* sequences obtained in the present study.

Sample ID	Query ID	Closest Match (NCBI accession)	% Identity	Query Coverage	E-value	Organism	Accession number
P1-D3-C1	lcl|Query_4812215	NG_047903.1	100%	100%	0.0	*S. ureilyticus*	PX828997
P1-D4-C1	lcl|Query_4873319	NG_047903.1	100%	100%	0.0	*S. kloosii*	PX828998
P2-D3-C1	lcl|Query_4929729	NG_047903.1	100%	100%	0.0	*S. kloosii*	PX828999
P3-D3-C1	lcl|Query_4972017	NG_047903.1	100%	100%	0.0	*S. ureilyticus*	PX829000
P3-D4-C1	lcl|Query_1522937	NG_047903.1	100%	100%	0.0	*S. kloosii*	PX829001
P7-D4-C2	lcl|Query_5043751	NG_047903.1	100%	100%	0.0	*S. ureilyticus*	PX829002

> P1-D3-C1_fusF_partial
TCTAAAATAGACATTTATCAGCAGTTTCATCAAATAGATGATACGTTGACTGAAGCGATTGAAAAATTAATGAACATACGTATTACTAAAGTACAAG
TAGATAAAATATTAGAAACTTTACAGACATACGTTATACCCTTTGAACACCCTTCAAAAAAACAAGTCGAAAAAACTTTTCGTAAAATTAAAAAGC
TTAAATCACCGCTTATTAGTGATGAAATCCTTTTGGAAAGTACTTATATCGGATGGAACGATATCGCTTCAAACAGAAAATTTATCATCTATTATAAC
GAGCAAGGAACTTTAACAGGTTTTTATGGCGACATTGCTAACCAAACCGTTAAGGGCTATTGCGCAATTTGTAATAAAGAATCTAATGTCGCTCTAT
TTATGCGTAAAACACGTACTTCTGGTGATGGACAATATACCAATT

> P1-D4-C1_fusF_partial
TCTAAAATAGACATTTATCAGCAGTTTCATCAAATAGATGATACGTTGACTGAAGCGATTGAAAAATTAATGAACATACGTATTACTAAAGTACAAG
TAGATAAAATATTAGAAACTTTACAGACATACGTTATACCCTTTGAACACCCTTCAAAAAAACAAGTCGAAAAAACTTTTCGTAAAATTAAAAAGC
TTAAATCACCGCTTATTAGTGATGAAATCCTTTTGGAAAGTACTTATATCGGATGGAACGATATCGCTTCAAACAGAAAATTTATCATCTATTATAAC
GAGCAAGGAACTTTAACAGGTTTTTATGGCGACATTGCTAACCAAACCGTTAAGGGCTATTGCGCAATTTGTAATAAAGAATCTAATGTCGCTCTAT
TTATGCGTAAAACACGTACTTCTGGTGATGGACAATATACCAAC
> P2-D3-C1_fusF_partial

TCTAAAATAGACATTTATCAGCAGTTTCATCAAATAGATGATACGTTGACTGAAGCGATTGAAAAATTAATGAACATACGTATTACTAAAGTACAAG
TAGATAAAATATTAGAAACTTTACAGACATACGTTATACCCTTTGAACACCCTTCAAAAAAACAAGTCGAAAAAACTTTTCGTAAAATTAAAAAGC
TTAAATCACCGCTTATTAGTGATGAAATCCTTTTGGAAAGTACTTATATCGGATGGAACGATATCGCTTCAAACAGAAAATTTATCATCTATTATAAC
GAGCAAGGAACTTTAACAGGTTTTTATGGCGACATTGCTAACCAAACCGTTAAGGGCTATTGCGCAATTTGTAATAAAGAATCTAATGTCGCTCTAT
TTATGCGTAAAACACGTACTTCTGGTGATGGACAATATACCAAC

> P3-D3-C1_fusF_partial
TCTAAAATAGACATTTATCAGCAGTTTCATCAAATAGATGATACGTTGACTGAAGCGATTGAAAAATTAATGAACATACGTATTACTAAAGTACAAG
TAGATAAAATATTAGAAACTTTACAGACATACGTTATACCCTTTGAACACCCTTCAAAAAAACAAGTCGAAAAAACTTTTCGTAAAATTAAAAAGC
TTAAATCACCGCTTATTAGTGATGAAATCCTTTTGGAAAGTACTTATATCGGATGGAACGATATCGCTTCAAACAGAAAATTTATCATCTATTATAAC
GAGCAAGGAACTTTAACAGGTTTTTATGGCGACATTGCTAACCAAACCGTTAAGGGCTATTGCGCAATTTGTAATAAAGAATCTAATGTCGCTCTAT
TTATGCGTAAAACACGTACTTCTGGTGATGGACAATATACCA

> P3-D4-C1_fusF_partial
TCTAAAATAGACATTTATCAGCAGTTTCATCAAATAGATGATACGTTGACTGAAGCGATTGAAAAATTAATGAACATACGTATTACTAAAGTACAAG
TAGATAAAATATTAGAAACTTTACAGACATACGTTATACCCTTTGAACACCCTTCAAAAAAACAAGTCGAAAAAACTTTTCGTAAAATTAAAAAGC
TTAAATCACCGCTTATTAGTGATGAAATCCTTTTGGAAAGTACTTATATCGGATGGAACGATATCGCTTCAAACAGAAAATTTATCATCTATTATAAC
GAGCAAGGAACTTTAACAGGTTTTTATGGCGACATTGCTAACCAAACCGTTAAGGGCTATTGCGCAATTTGTAATAAAGAATCTAATGTCGCTCTAT
TTATGCGTAAAACACGTACTTCTGGTGATGGACAATATACCAAC

> P7-D4-C2_fusF_partial
TCTAAAATAGACATTTATCAGCAGTTTCATCAAATAGATGATACGTTGACTGAAGCGATTGAAAAATTAATGAACATACGTATTACTAAAGTACAAG
TAGATAAAATATTAGAAACTTTACAGACATACGTTATACCCTTTGAACACCCTTCAAAAAAACAAGTCGAAAAAACTTTTCGTAAAATTAAAAAGC
TTAAATCACCGCTTATTAGTGATGAAATCCTTTTGGAAAGTACTTATATCGGATGGAACGATATCGCTTCAAACAGAAAATTTATCATCTATTATAAC
GAGCAAGGAACTTTAACAGGTTTTTATGGCGACATTGCTAACCAAACCGTTAAGGGCTATTGCGCAATTTGTAATAAAGAATCTAATGTCGCTCTAT
TTATGCGTAAAACACGTACTTCTGGTGATGGACAATATACCAAC

Table S3. Information about the BLAST identity of the partial 16S rRNA sequences obtained in the present study.

**Sample ID**	**Query ID**	**Closest Match (NCBI accession)**	**% Identity**	**Query Coverage**	**E-value**	**Organism**	**Accession number**
P3-D3-C1	lcl|Query_6253551	JX102547.1	99.93%	100%	0.0	*S. ureilyticus*	PX765221
P3-D4-C1	lcl|Query_4668957	MW365210.1	100%	100%	0.0	*S. kloosii*	PX765222
P7-D4-C2	lcl|Query_5861631	MW365210.1	100%	100%	0.0	*S. ureilyticus*	PX765223

> P3-D3-C1_16S_rRNA_partial
ATACATGCAGTCGAGCGACAGATAAGGAGCTTGCTCCTTTGACGTTAGCGGCGGACGGGTGAGTAACACGTGGGTAACCTACCTATAAGACTGGAA
TAACTCCGGGAAACCGGGGCTAATGCCGGATAACATTTAGAACCGCATGGTTCTAAAGTGAAAGATGGTTTTGCTATCACTTATAGATGGACCCGCG
CCGTATTAGCTAGTTGGTAAGGTAACGGCTTACCAAGGCAACGATACGTAGCCGACCTGAGAGGGTGATCGGCCACACTGGAACTGAGACACGGTC
CAGACTCCTACGGGAGGCAGCAGTAGGGAATCTTCCGCAATGGGCGAAAGCCTGACGGAGCAACGCCGCGTGAGTGATGAAGGTCTTCGGATCGT
AAAACTCTGTTATTAGGGAAGAACAAATGTGTAAGTAACTGTGCACGTCTTGACGGTACCTAATCAGAAAGCCACGGCTAACTACGTGCCAGCAGC
CGCGGTAATACGTAGGTGGCAAGCGTTATCCGGAATTATTGGGCGTAAAGCGCGCGTAGGCGGTTTCTTAAGTCTGATGTGAAAGCCCACGGCTCA
ACCGTGGAGGGTCATTGGAA

>P3-D4-C1_16S_rRNA_partial
ATGCAGTCGAGCGACAGATAAGGAGCTTGCTCCTTTGACGTTAGCGGCGGACGGGTGAGTAACACGTGGGTAACCTACCTATAAGACTGGAATAAC
TTCGGGAAACCGAAGCTAATGCCGGATAACATGTAGAACCGCATGGTTCTACAGTGAAAGATGGCCTTGCTATCACTTATAGATGGACCCGCGCCG
TATTAGCTAGTTGGTAAGGTAACGGCTTACCAAGGCAACGATACGTAGCCGACCTGAGAGGGTGATCGGCCACACTGGAACTGAGACACGGTCCAG
ACTCCTACGGGAGGCAGCAGTAGGGAATCTTCCGCAATGGGCGAAAGCCTGACGGAGCAACGCCGCGTGAGTGATGAAGGTCTTCGGATCGTAAA
GCTCTGTTATTAGGGAAGAACAAGTGCGTAAGTAACTGTGCGCACCTTGACGGTACCTAATCAGAAAGCCACGGCTAACTACGTGCCAGCAGCCGC
GGTAATACGTAGGTGGCAAGCGTTATCCGGAATTATTGGGCGTAAAGCGCGCGTAGGCGGTTTCTTAAGTCTGATGTGAAAGCCCACGGCTCAACC
GTGGAGGGTCATTGGAAACTGGGAGACTTGAGTGCAGAAGAGGAAAGTGGAATTCCATGTGTAGCGGTGAAATGCGCAGAGATATGGAGGAACAC
CAGTGGCGAAGGCGACTTTCTGGTCTGTAACTGACGCTGATGTGCGAAAGCGTGGGGATCAAACAGGATTAGATACCCTGGTAGTCCACGCCGTAA

ACGATGAGTGCTAAGTGTTAGGGGGTTTCCGCCCCTTAGTGCTGCAGCTAACGCATTAAGCACTCCGCCTGGGGAGTACGACCGCAAGGTTGAAACT
CAAAGGAATTGACGGGGACCCGCACAAGCGGTGGAGCATGTGGTTTAATTCGAAGCAACGCGAAGAACCTTACCAAATCTTGACATCCTTTGACCA
CTCTGGAGACAGAGTTTTCCCCTTCGGGGGACAAAGTGACAGGTGGTGCATGGTTGTCGTCAGCTCGTGTCGTGAGATGTTGGGTTAAGTCCCGCAA
CGAGCGCAACCCTTAAGCTTAGTTGCCATCATTAAGTTGGGCACTCTAAGTTGACTGCCGGTGACAAACCGGAGGAAGGTGGGGATGACGTCAAAT
CATCATGCCCCTTATGATTTGGGCTACACACGTGCTACAATGGACAATACAAAGGGCAGCTAAACCGCGAGGTCATGCAAATCCCATAAAGTTGTT
CTCAGTTCGGATTGTAGTCTGCAACTCGACTACATGAAGCTGGAATCGCTAGTAATCGTAGATCAGCATGCTACGGTGAATACGTTCCCGGGTCTTGT
ACACACCGCCCGTCACACCACGAGAGTTTGTAACACCCGAAGCCGGTGGAGTAACCATTATGGAGCTA

>P7-D4-C2_16S_rRNA_partial
ATACATGCAGTCGAGCGACAGATAAGGAGCTTGCTCCTTTGACGTTAGCGGCGGACGGGTGAGTAACACGTGGGTAACCTACCTATAAGACTGGAA
TAACTCCGGGAAACCGGGGCTAATGCCGGATAACATTTAGAACCGCATGGTTCTAAAGTGAAAGATGGTTTTGCTATCACTTATAGATGGACCCGCG
CCGTATTAGCTAGTTGGTAAGGTAACGGCTTACCAAGGCAACGATACGTAGCCGACCTGAGAGGGTGATCGGCCACACTGGAACTGAGACACGGTC
CAGACTCCTACGGGAGGCAGCAGTAGGGAATCTTCCGCAATGGGCGAAAGCCTGACGGAGCAACGCCGCGTGAGTGATGAAGGTCTTCGGATCGT
AAAACTCTGTTATTAGGGAAGAACAAATGTGTAAGTAACTGTGCACGTCTTGACGGTACCTAATCAGAAAGCCACGGCTAACTACGTGCCAGCAGC
CGCGGTAATACGTAGGTGGCAAGCGTTATCCGGAATTATTGGGCGTAAAGCGCGCGTAGGCGGTTTCTTAAGTCTGATGTGAAAGCCCACGGCTCA
ACCGTGGAGGGTCATTGGAAACTGGGAAACTTGAGTGCAGAAGAGGAAAGTGGAATTCCATGTGTAGCGGTGAAATGCGCAGAGATATGGAGGAA
CACCAGTGGCGAAGGCGACTTTCTGGTCTGTAACTGACGCTGATGTGCGAAAGCGTGGGGATCAAACAGGATTAGATACCCTGGTAGTCCACGCCG
TAAACGATGAGTGCTAAGTGTTAGGGGGTTTCCGCCCCTTAGTGCTGCAGCTAACGCATTAAGCACTCCGCCTGGGGAGTACGACCGCAAGGT

Table S4. Information on the nucleotide sequences of the fusidic acid resistant genes retrieved from GenBank database used in the construction of the phylogenetic tree.

**Species**	**Fusidic acid resistant gene**	**Accession number**	**Role in the phylogenetic tree**
S. ureilyticus	*fusF*	NG_047903.1	Control (1)
S. ureilyticus	*fusF*	NG_047904.1	Control (2)
S. aureus	*fusC*	NG_050413.1	Outgroup
